# Metabolomic Profiling of the Secretome from Human Neural Stem Cells Flown into Space

**DOI:** 10.3390/bioengineering11010011

**Published:** 2023-12-22

**Authors:** Juan Carlos Biancotti, Araceli Espinosa-Jeffrey

**Affiliations:** 1 Department of Surgery/Pediatric Surgery, School of Medicine, Johns Hopkins University, Baltimore, MD 21205, USA; jbianco5@jhmi.edu; 2 Department of Psychiatry, IDDRC, Semel Institute, University of California Los Angeles, Los Angeles, CA 90095, USA

**Keywords:** microgravity, neural stem cells, secretome, spaceflight, metabolomics, cell metabolism

## Abstract

The change in gravitational force has a significant effect on biological tissues and the entire organism. As with any alteration in the environment, microgravity (µG) produces modifications in the system inducing adaptation to the new condition. In this study, we analyzed the effect of µG on neural stem cells (NSCs) following a space flight to the International Space Station (ISS). After 3 days in space, analysis of the metabolome in culture medium revealed increased glycolysis with augmented pyruvate and glycerate levels, and activated catabolism of branched-chain amino acids (BCAA) and glutamine. NSCs flown into space (SPC-NSCs) also showed increased synthesis of NADH and formation of polyamine spermidine when compared to ground controls (GC-NSCs). Overall, the space environment appears to increase energy demands in response to the µG setting.

## 1. Introduction

The increasing duration of space missions and consequent need to adapt to the inhospitable conditions of the space environment present new challenges to astronauts. The health and safety of present and future space travelers remains paramount. Long periods of exposure to µG may have detrimental effects on tissues and their regenerative potential, partly due to changes observed in stem cell populations [1,2]. In particular, the human brain suffers alterations in the structure and electrocortical function induced by microgravity (µG). These alterations manifest when the space crew returns to Earth as problems with motor coordination increase intracranial pressure, and a vestibular system containing otoliths is unable to sense the presence of gravity [3,4,5]. At the cellular level, Silvano et al. reported transient cell cycle arrest and cellular stress followed by apoptosis on murine cerebellar neural stem cells (NSCs). The cells recovered their normal cell cycle and stemness upon returning to normal gravity [6]. Our own studies and those from other groups have shown an increase in cell proliferation and metabolic activity of human NSCs on a spaceflight or under simulated µG (sim- µG), in part due to an increase in mitochondrial function [7,8]. Mattei et al. studied early brain development under simulated µG using human embryonic stem cell-derived neural organoids. Although the organoids were able to form, they reported alterations in the rostral-caudal patterning, indicating that µG favors differentiation toward caudal neural progenitors [9].

The impact of a lack of gravitational force on biological systems can be observed soon after the exposure begins. After a 24 h incubation in a sim-µG, a human seminoma cell line exhibited markers of oxidative stress, such as enlarged mitochondria and increased intracellular ROS, protein and lipid oxidation, and SOD [10]. Human gastric mucosal cells showed signs of stress and inflammation following 3 days of sim-µG, whereas oxidative stress on the same cells was detected after 7 days of exposure to microgravity [11]. Our own studies on fetal human NSC-derived oligodendrocyte progenitors (OLPs) revealed increased mitochondrial respiration and increased glycolysis after 24 h of exposure to sim-µG. Moreover, examination of the secretome after exposing OLPs to sim-µG for 3 days increased the TCA cycle and the synthesis of fatty acids and complex lipids [12]. A more detailed study on mitochondrial function in rat hippocampus was performed by Wang et al., following 7 days in a tail suspension model of microgravity. They found downregulation in mitochondrial complexes I, III, and IV, in isocitrate and malate dehydrogenases, as well as upregulation of the antioxidant enzymes PRX6 and DJ-1 [13].

In this study, we sought to ascertain “gravity sensitive” small molecules secreted by NSCs after short-term exposure to space µG. Our results demonstrate that µG increases energy production, mainly through the TCA cycle, and triggers stress response mechanisms as means for adaptation.

## 2. Materials and Methods

### 2.1. Cells and Space Flight Information

**Figure 1:**
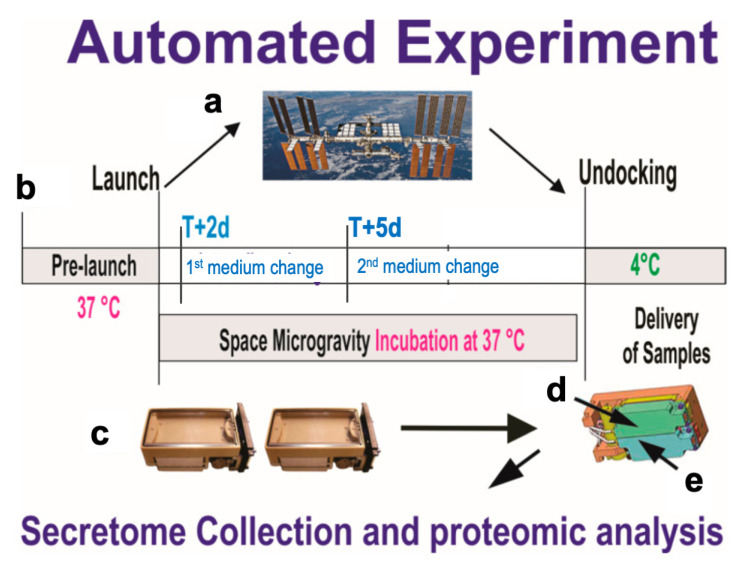
Synopsis of the automated experiments. (**a**) On board SpaceX-16. (**b**) Timeline displaying initial media change at T + 2 d after NSCs reached space. After 3 days (T + 5 d), the second culture medium was recovered into the second tank just prior to unberth, and stored at 4 °C. This conditioned medium contains the molecules secreted solely while cells were onboarding the ISS. During ascent, descent, and space flight, cells were maintained at 37 °C. Upon arrival to our laboratory, the culture media were recovered separately and placed in numbered tubes with the addition of a cocktail of proteases inhibitors, and saved frozen at −80 °C. (**c**) View of the cell chamber, where mesh carriers with cells and the travel culture medium travelled. (**d**,**e**) Tanks that contained the fresh medium to be released at T + 2 to start the experiment. Tank 2 (E arrow) contained the second fresh medium with which the cells came back to Earth. There was not a 1 G control onboard the space station, as tests were performed two years prior to determine the optimal conditions for the cells. The STaARS-1 EF facility was created by STaARS, inspired by this study to reduce the time astronauts would have to invest in these experiments. This facility was activated, monitored, and controlled from STaARS’ headquarters in Houston, TX. We used a total of four units that were flown aboard SpaceX-16 on 5 December 2018. Image adapted from Vergnes et al. [16].

A homogeneous population of NSCs was obtained from human-induced pluripotent stem cells (hiPS). The original cells, known as “CS83iCTR-33nxx” (such as skin cells), were “reprogrammed” and provided to us by Cedars-Sinai Medical Center via a material transfer agreement, flown to the International Space Station (ISS), and installed in the Space Technology and Advanced Research System Experiment Facility-1 at 37 °C. NSCs were seeded using STM culture medium designed in our laboratory [14]. For the space flight, NSCs were seeded onto mesh carriers (2 mm × 3 mm) and placed inside a Type IV cell culture chamber. They were flown to the International Space Station (ISS) and installed in the Space Technology and Advanced Research System Experiment Facility-1 at 37 °C. We used the Automated Type IV chamber intended to collect the secretome of NSCs for 3 days while in space to determine their proliferation by BrdU incorporation (Figure 1). After 3 days of incubation, the culture medium was pumped out of the cell chamber, and the units were stored at 4 °C. After splash-down, samples were transported in a controlled environment. Upon arrival to UCLA, the NSCs were retrieved from the hardware, plated onto poly-d-lysine coated flasks in our proprietary stem cell chemically defined medium (STM) [15], and allowed to recover from space flight and splash-down.

### 2.2. Secretome Collection

The culture medium that fed cells during the 3 distinct time points (during the space flight, during the 3 day exposure on the ISS, and following the 3 day exposure but prior to splash-down) were recovered and saved frozen at −80 °C. This medium is commonly known as the conditioned medium and contains proteins, peptides, metabolites, and many more products usually secreted by the cells into the medium. For the purpose of this manuscript, we named this conditioned medium the “secretome”. We use the term “space CM” in comparative figures to differentiate this from conditioned medium derived from ground control or naïve cells.

### 2.3. Metabolomic Profile of the Secretome

The metabolic profile was obtained for each group using the Metabolon Platform (Metabolon). Briefly, each sample or “group” consisted of 5 biological replicate specimens. Three groups of samples were compared: blank (*n* = 5), which consisted of the culture medium alone, incubated in the same incubator used for our cell cultures; NSCs (*n* = 5) in 1 G; and NSCs incubated in SPC-flown NSCs (*n* = 5). The secretome was subjected to methanol extraction, then split into aliquots for analysis by ultrahigh performance liquid chromatography/mass spectrometry (UHPLC/MS) in the positive (two methods), negative, or polar ion mode. Metabolites were identified by automated comparison of ion features to a reference library of chemical standards, followed by visual inspection for quality control, as previously described [17]. Random forest analysis was performed as described [18,19]. For the box plots, whiskers reflect the 5th and 95th percentiles (with the box displaying the 25th to 75th quartiles); the bisecting line represents the population median, while “+” indicates the population mean [16]. For this study, we considered solely the molecules that presented a significant difference (*p* < 0.05) between groups, with a metabolite ratio of <1.00. The metabolomics study, compound identification, and statistical analysis were performed as recently published [20]. We used groups of *n* = 5. For SPC, 1 G, and control medium, Welsh’s two-sample t-tests were performed with ArrayStudio (Omicsoft) or “R” to compare between the data; *p* < 0.05 was considered significant (Array Studio, RRID:SCR_010970). The false discovery rate (Q-value) was calculated, taking into account the multiple comparisons that normally occur in metabolomic-based studies (Q < 0.05), and was used as an indication of high confidence in the data. Both Hierarchical Clustering Analysis (HCA) and Principal Component Analysis (PCA) were also performed in ArrayStudio.

## 3. Results

### 3.1. Random Forest and Hierarchical Cluster Analysis

We performed metabolomic analysis on the culture medium from control Earth-grounded NSCs (GC-NSCs; 1 G) and space-flown NSCs (SPC-NSCs; 0 G). The 3D “secretome” (conditioned culture medium) was produced and collected for 3 days, starting on T + 2 and concluding on T + 5, as illustrated in Figure 1. Therefore, it contains solely the molecules secreted by NSCs while in space (active experiment). A control for changes that may occur in the culture medium under 1 G conditions was added to the analysis.

**Figure 2:**
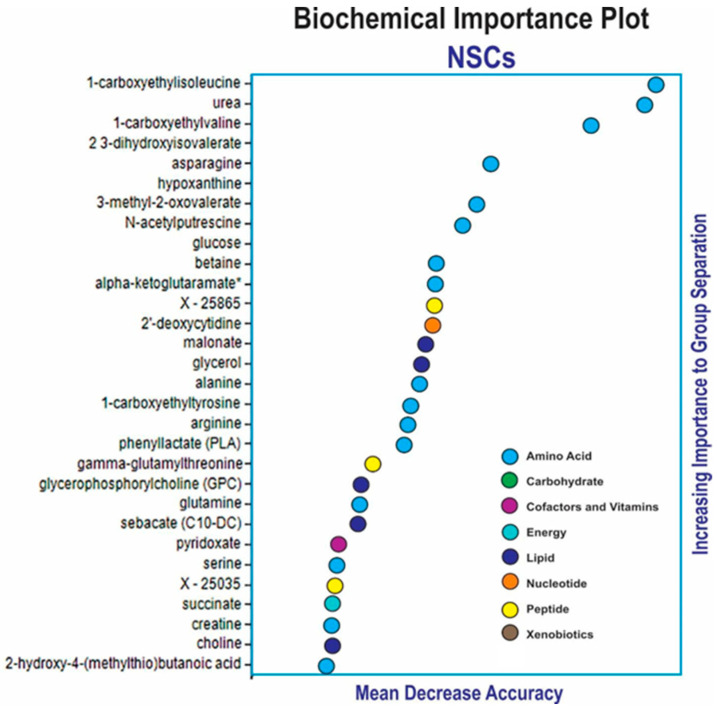
Random forest analysis (RFA). The assessment of blank, ground control, and SPC-flown NSCs was highly effective. The biochemical importance plot highlighted biochemicals related to a number of super family pathways: amino acids associated with glutaminolysis (Ala, Asn, Asp, Gln), amino acid metabolites from BCAA catabolism (1-carboxyethylisoleucine, 1-carboxyethylvaline, 2,3-dihydroxyisovalerate, 3-methyl-2-oxovalerate, alpha-ketoglutaramate), metabolites from nitrogen metabolism (urea, creatine, N-acetyl-putrescine), lipids associated with metabolism of phospholipids (malonate, glycerol, choline, glycerophosphorylcholine), and nucleotide metabolism (hypoxanthine, 2′-deoxycytidine). Random forest classification using named metabolites in media of blank compared to 1 G and 0 G NSCs gave a predictive accuracy of 100%. Random chance would be expected to yield a predictive accuracy of 33%. We found two molecules with unclear identities: X25035, which is most likely a peptide, and X25865. RFA was performed as previously described [20].

In order to gain insight into the potential biomarkers produced by NSCs under the conditions of space flight vs. ground control, we used the Random Forest Analysis (RFA) approach, which uses a forest of decision trees to identify molecules that are important to predict groups. The RFA assessment of blank, ground control, and SPC-flown NSCs was very effective. The biochemical importance plot highlighted a number of amino acids and their catabolites related to glutaminolysis (Ala, Asp, Gln, alpha-ketoglutaramate), BCAA catabolism (1-carboxyethylisoleucine, 1-carboxyethylvaline, 2,3-dihydroxyisovalerate, 3-methyl-2-oxovalerate), nitrogen balance (urea, creatine, N-acetyl-putrescine), glucose and succinate involved in energy production, and lipids and lipid precursors (glycerol, choline, glycerophosphorylcholine) related to the synthesis of phospholipids and cellular membranes (Figure 2).

### 3.2. NSCs in Space (SPC-NSCs) Have High Energy Demands

**Figure 3:**
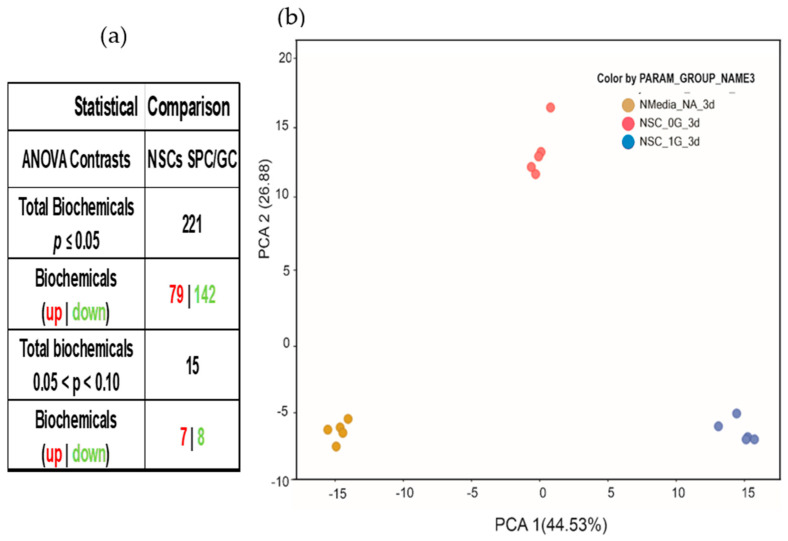
Global biochemical changes. (**a**) The statistical comparison of metabolites secreted by NSCs during the 3 days aboard the ISS vs. those secreted by GC-NSCs revealed a total of 221 biochemical alterations, of which 79 increased and 142 decreased. This secretome was produced and collected over 3 days, starting on T + 2 and concluding on T + 5, using the automated hardware. Therefore, it contains only those molecules secreted during the 3 days onboard the ISS. Only statistically significant species were considered in the analysis. (**b**) Principal component analysis (PCA) demonstrates that each of the three sample groups (NSC-SPC [red], NSC-GC [blue], and control medium [yellow]) have a different biochemical profile of its secretome after 3 days, based on its gravitational status.

A total of 221 biochemicals were identified, with significantly different levels of expression between the NSCs-SPC and NSCs-GC secretomes. From them, 79 were increased and 142 were decreased in NSCs-SPC, with respect to NSCs-GC (Figure 3a). Principal Component Analysis (PCA) of this data-set revealed three distinct groups of samples clustered together with a clear separation between them, indicating different biochemical profiles in each group’s secretome (Figure 3b).

**Figure 4:**
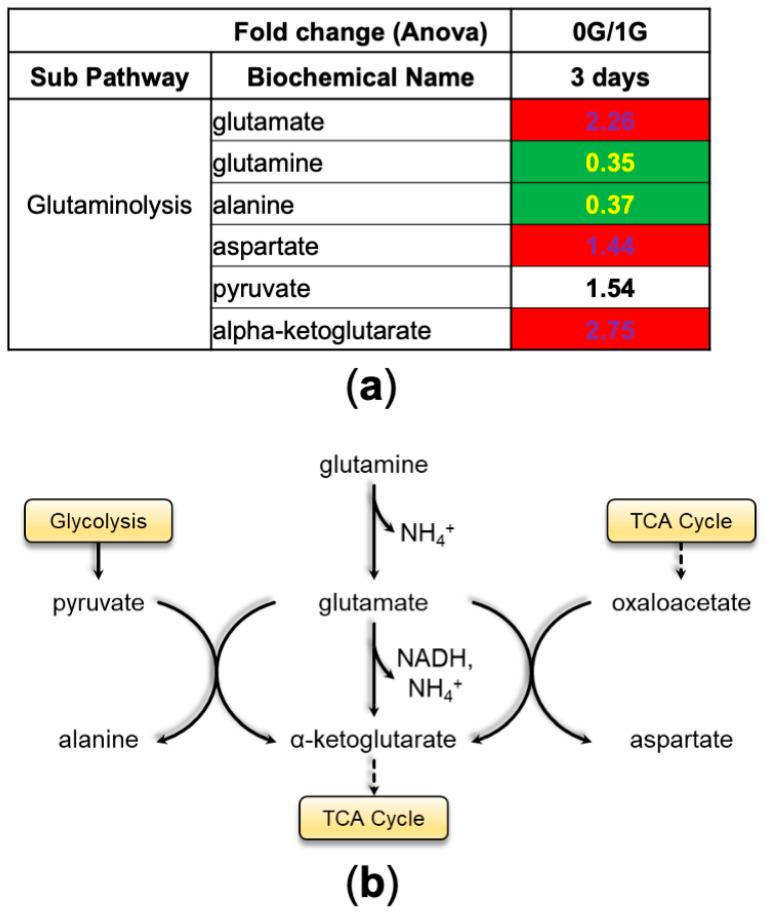
Glutaminolysis. (**a**) Table displaying the fold change ratio of glutamine and its metabolites after spending 3 days in space (0 G) vs. ground control (1 G). Values in red rectangles represent increased levels of the metabolites in space compared to ground control, whereas green rectangles highlight the opposite and white rectangles represent no change. (**b**) Scheme of the glutamine metabolic pathway.

**Figure 5:**
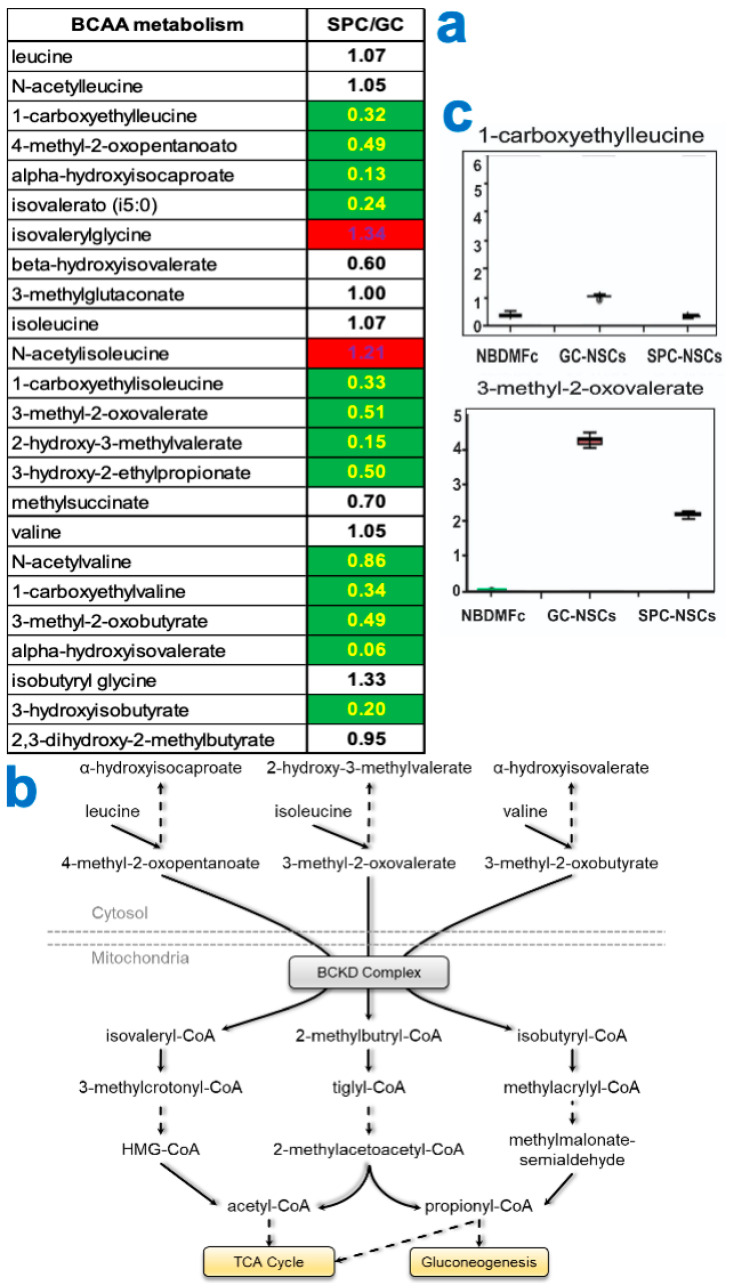
BCAA catabolism. (**a**) Table depicting the fold change ratio of the 3 BCAAs, leucine, isoleucine, and valine, and their metabolites after 3 days in space (SPC) or ground control (GC). Values in red rectangles represent increased levels of the metabolites in space compared to ground control, whereas green rectangles highlight the opposite and white rectangles represent no change. (**b**) Scheme of the BCAA metabolic pathways. (**c**) Example of the levels of 2 metabolites.

By grouping biochemicals by metabolic pathways, we found increased levels of glutamate and alpha-ketoglutarate and a concomitant reduction in glutamine and alanine aminoacids, which indicates an increase in glutaminolysis to provide substrates to the Tri-Carboxylic Acid Cycle (TCA) for generation of energy (Figure 4). We also observed increased catabolism of branched-chain amino acids (BCAA) and an overall reduction in mitochondrial and cytoplasmic intermediate metabolite levels at 0 G compared to cells that remained at 1 G; Tthe reduction was most evident in the cytoplasmic levels. These findings, along with the fact that levels of BCAA were similar between the two conditions, suggest recent activation of BCAA catabolism (Figure 5a). BCAA catabolism leads to the production of acetyl-CoA, which enters the TCA cycle to generate energy, and propionyl-CoA, which can be directed to either the TCA cycle or the gluconeogenesis pathway. In both cases, the main aim of BCAA catabolism is the production of energy (Figure 5b).

**Figure 6:**
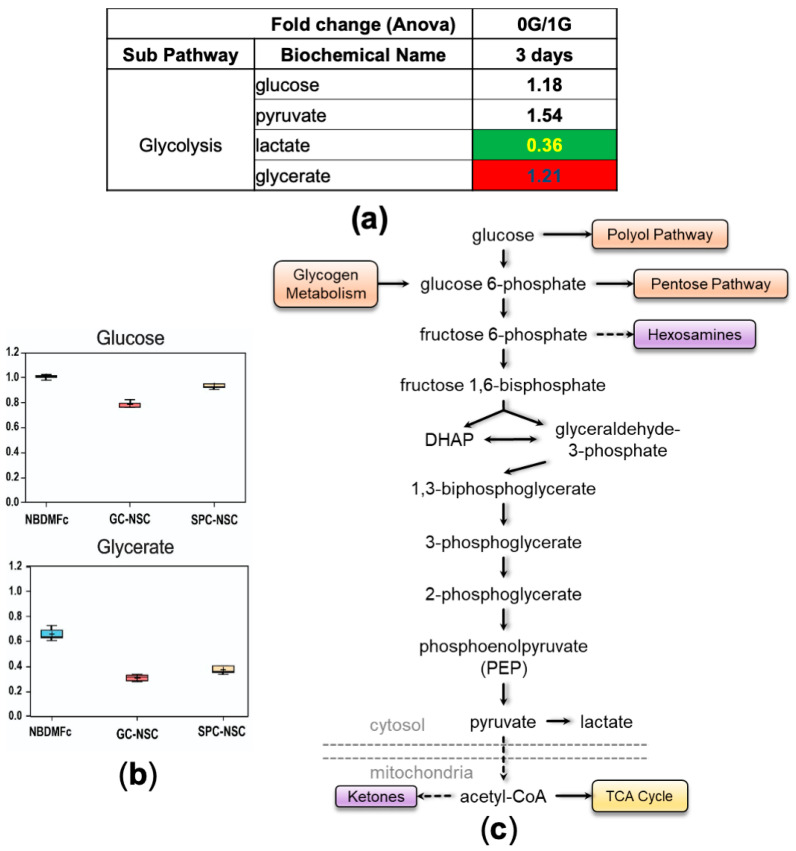
Glycolysis. (**a**) Table displaying fold change ratio of the glycolytic pathway metabolites at 3 days of 0 G vs. 1 G NCS secretome. Values in red rectangles represent increased levels of the metabolites in space compared to ground control, whereas green rectangles highlight the opposite and white rectangles represent no change. (**b**) Example of 2 metabolites levels. (**c**) Scheme of the glycolytic pathway.

We then looked at the status of the glycolytic pathway. Higher levels of pyruvate accompanied by lower levels of lactate suggested that cells preferentially utilized the glycolytic pathway for a more efficient TCA energetic pathway (Figure 6), or that lactate was used as an oxidative substrate for energy metabolism [21]. These observations are in concert with the activation of amino acids’ catabolism as a supplementary source of energy, which may indicate that NSCs-SPC require more energy to adapt to 0 G conditions.

**Figure 7:**
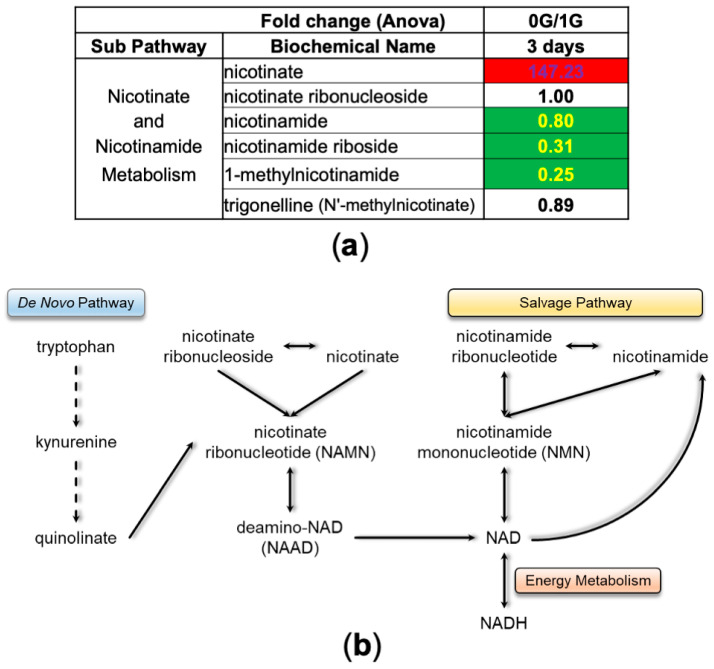
NADH synthesis. (**a**) Table depicting fold change ratio of intermediates of the NADH synthesis pathway intermediates from NSC-SPC (0 G) vs. NSC-GC (1 G) after 3 days of incubation. Values in red rectangles represent increased levels of the metabolites in space compared to ground control, whereas green rectangles highlight the opposite and white rectangles represent no change. (**b**) Scheme of the “de novo” and “salvage” pathways.

The NAD^+^/NADH system is an important player in energy metabolism, accumulating energy by reduction of NAD^+^ and releasing it by oxidation of NADH. Analysis of metabolic intermediates in the synthesis of NAD^+^ showed that 3 days in space strongly stimulated the synthesis of NAD^+^ through a de novo pathway as opposed to the salvage pathway, as visualized by a marked increase in nicotinate and reduction in nicotinamide intermediates (Figure 7).

**Figure 8:**
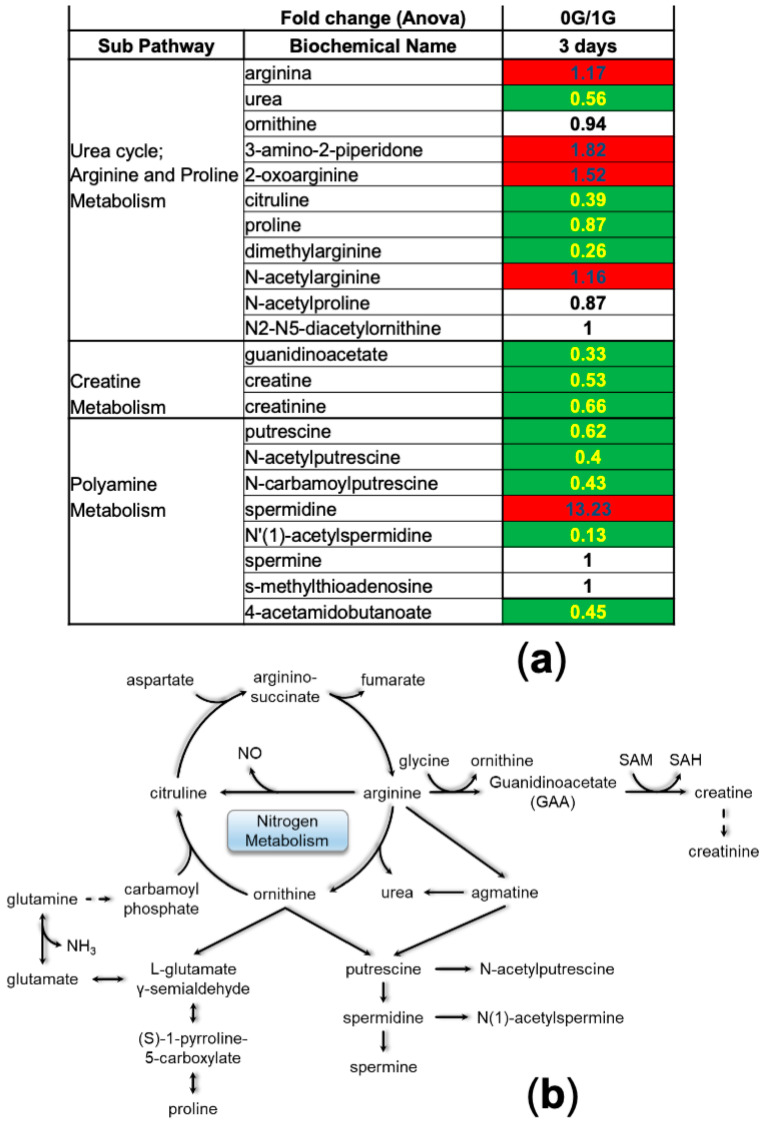
Nitrogen Metabolism. (**a**) Table depicting the fold change ratio of nitrogen metabolism intermediates between 0 G vs. 1 G secretomes after 3 days of incubation. Nitrogen metabolites are divided into urea cycle, creatine metabolism, and polyamine metabolism pathways. Values in red rectangles represent increased levels of the metabolites in space compared to ground control, whereas green rectangles highlight the opposite and white rectangles represent no change. (**b**) Scheme of the integrated nitrogen metabolism.

Amino acid metabolism generates nitrogen waste metabolites. Several fates for this nitrogen exist, including incorporation into creatine or polyamines, transport to the liver via glutamine, and the subsequent production of urea. The levels of creatine, creatinine and urea are reduced in NSCs-SPC compared to NSCs-GC, despite the increased metabolism of amino acids. However, there is a significant increase in the levels of these metabolites after 39 days in space (unpublished results), which suggests that we are witnessing the beginning of the adaptive mechanism to 0 G. Interestingly, polyamine metabolism revealed a significant increase in spermidine, while its precursor putrescine and its metabolites acetylated for degradation—N-acetyl-putrescine and N-acetyl-spermidine—remained low (Figure 8). Considering the involvement of spermidine in cell homeostasis and particularly in autophagy, the increased levels after only 3 days in space may reveal another adaptive mechanism to cope with the stress imposed by low gravity.

**Figure 9:**
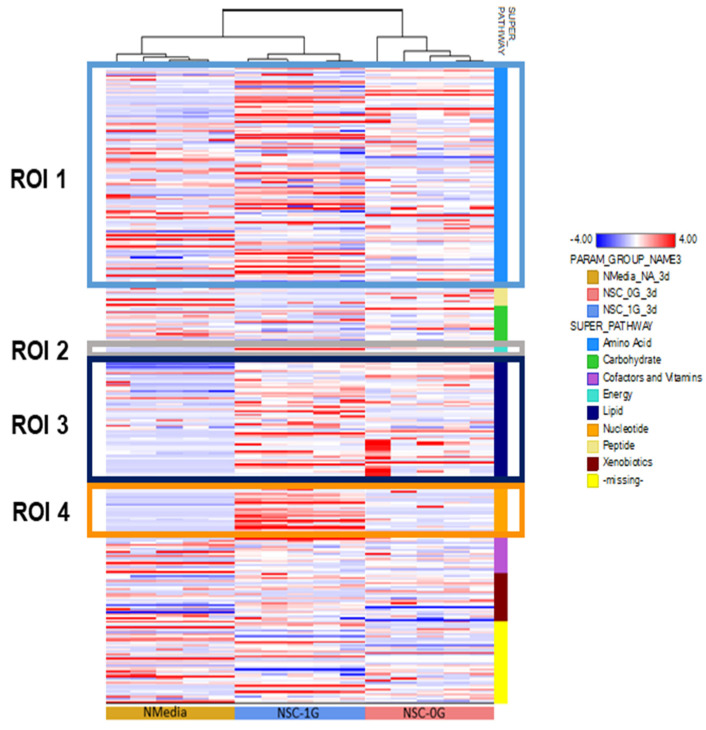
The hierarchical cluster analysis (HCA) evaluates the variable clustering patterns of the metabolites from space--lown NSCs and ground control. We show the data in a table format, where the rows represent individual metabolites and the columns represent the secretome sample from which molecules were detected: STM medium (**yellow**), GC-NSCs (**blue**), and SPC-NSCs (**red**). The color of the cell reflects the concentration of each metabolite to its corresponding sample, relative to the mean concentration level across the entire set of tissue samples. Respectively, the colors red, blue, and white within the table indicate an increase, decrease, or no significant change of metabolites. The pathways that the metabolites belong to are indicated to the right of the HCA. The HCA compares the metabolite change between the 0 G, 1 G, and control groups. *n*  =  5 samples per group. HCA data analysis was generated using ArrayStudio.

The hierarchical cluster analysis (HCA) evaluates the variable clustering patterns of the metabolites from space-flown NSCs, compared to the ground control. In addition to several amino acids (and related metabolites), the TCA metabolites alpha-ketoglutarate and succinate were higher in NSCs-SPC, consistent with an increase in energy production as discussed above. There was also a reduction in phospholipids (phosphoethanolamine, glycerophosphorylcholine) and ketone bodies (3-hydroxybutirate), with an increase in glycerol-3-phosphate, suggesting that lipids may be used as a source for energy in addition to glucose and amino acids. In the nucleotide area, there was an overall reduction in nitrogen bases and metabolic intermediaries (N-methyl adenosine, orotate, xanthine) (Figure 9).

## 4. Discussion

The effect of microgravity on the human body has been increasingly studied over the past several decades, in part due to the higher frequency of space trips, increased duration of space missions, and planning for future space exploration involving long-term flights with humans. The change in gravitational forces, as with any modification in environmental conditions, induces adaptive responses in living organisms. Understanding the gravitational biology of specific cells and organs is essential for successful travel and long-term survival in space. A comprehensive multi-Omics study from NASA’s GeneLab analyzed tissue samples from astronauts, with data pointing to mitochondrial dysfunction as the primary and consistent alteration caused by space travel [22]. Systemic alteration in mitochondrial function causes leaking of ROS and a reduction in antioxidant defenses, leading to increased oxidative stress, which accounts for most of the disorders affecting astronauts’ health [23].

Our aim is to investigate the adaptive changes that Central Nervous System (CNS) cells experience in a µG environment. In this work we focused on neural stem cells (NSCs), which have the potential to differentiate into various cell types, and contribute to maintaining the integrity and function of the nervous system in health and disease. Our previous work reported an increased proliferation of NSCs following space travel, which was accompanied by higher oxygen consumption and extracellular acidification rates, indicating increased metabolic activity of the cells in space compared to control NSCs grounded on Earth [7]. Here, we present evidence supporting an increase in the production and consumption of energy through a metabolomic analysis of the cell culture medium. Changes in the levels of intermediates of glycolysis, such as increases in pyruvate and glycerate in the SPC-NSC culture medium, are indicators of activated glycolysis. This activates the TCA cycle for more efficient aerobic energy production, as lactate levels declined significantly in 1 G-NSCs. In line with our findings, Chiang and collaborators exposed human NSCs to simulated µG and found an increase in cell proliferation, supported by enhanced mitochondrial function [8]. In another work, cultures of human biliary stem cells in simulated µG (sim- µG) also displayed activation of the glycolytic pathway, although with the production of fermentation and ketosis byproducts [24]. We have previously reported that simulated µG triggered increased mitochondrial respiration and increased glycolysis in another CNS population, oligodendrocyte progenitors (OLPs), as soon as 24 h after exposure to sim-μG. Moreover, examination of the secretome after 3 days of exposure of OLPs to sim-μG confirmed increased activity in the TCA cycle [25] flux in sim-μG [26]. It appears that soon after being exposed to sim-μG, cells have an acute increase in their need for energy, stimulating glycolysis and leading toward the less efficient anaerobic path, with higher lactic acid production. As the energy demands remain high, the glycolytic pathway transitions toward the TCA aerobic pathway.

In the present study, another indication of the increase in demand and production of energy elicited by µG is demonstrated by the active catabolism of BCAA observed in SPC-NSC, as the end products acetyl-CoA or propionyl-CoA also serve as substrates for the TCA cycle. Furthermore, the amino acid glutamine renders alpha-ketoglutarate, a TCA intermediary that is poured into the cycle to increase energy production. As more energy is required by cells in µG, more NAD is needed, as the NAD/NADH system is an essential player in energy metabolism. Energy is necessary for all cellular processes and NAD^+^ plays a key role as an effector substrate for sirtuins, PARPs, and CD38 which trigger signaling processes to maintain tissue physiology under environmental stresses [27]. As subproducts of amino acid catabolism, the polyamine spermidine plays a critical role in the maintenance of cellular homeostasis and is involved in cell growth and proliferation, tissue regeneration, DNA and RNA stabilization, enzymatic modulation, and regulation of translation, among others [28,29,30]. More importantly, it induces autophagy through inhibition of several acetyltransferases, including EP300 [31,32]. Via time-lapse microscopy, we observed significant activation of autophagy-like behavior in our cells upon return from space, and by proteomic analysis of NSCs-SPC secretome (manuscript submitted for publication). However, we have exhausted our current samples, and would require a second space flight in order to elucidate whether the phenomenon observed is indeed autophagy. The occurrence of autophagy has also been described in ARPE19 retina cells exposed to sim-μG, where an increase in the production of ROS and mitochondria dysfunction led to the activation of autophagy, and mitophagy [24].

Most of the studies on short-term exposure to microgravity were carried out on sim-µG on the Earth’s surface. Our study was carried out onboard the ISS, and samples were also exposed to all the stresses a biological system experiences during the space flight. However, one limitation of this study is the lack of a NSC culture at 1 G onboard the ISS to control for stress from takeoff and travel, as well as for ionizing radiation. Ionizing Radiation (IR) and μG exposure act synergistically in space, producing interactions between adaptive pathways and defense responses [33]. IR generates ROS/RNS, giving rise to oxidative stress and damage [1,34]. This poses a risk for the CNS, due to its high sensitivity to oxidative injury in the context of elevated levels of oxidizable, unsaturated lipids, and low levels of antioxidant defenses. In a mouse spleen model of low dose radiation exposure, γ-radiation, or high energy particle radiation, the authors observed enrichment of metabolites involved in purine metabolism, TCA, fatty acids, acylcarnitine, and amino acids. Early perturbations were more prominent in the γ irradiated samples, while later responses shifted towards more prominent responses in groups with high energy particle irradiations, suggesting a mitochondrial implication and possible long-term effects on DNA repair [35]. The added effect of IR to sim-µG was reported by Mao et al. in a study performed in mice brains exposed to either low-dose γ-radiation or hindlimb suspension (model for sim-µG), or to both conditions, for 21 days. Higher levels of oxidative stress and NADP oxidase 2, and lower levels of SOD were observed in the combined treatment group [35]. The potential influence of IR on the NSCs in our study is likely mitigated by the short-term exposure of the NSCs to IR and the protection that the Earth’s magnetic field exerts against Solar Particle Events, since the ISS is in a low Earth orbit, although it does not shield from Galactic Cosmic Radiation, which is composed of high-charge and energy particles.

In conclusion, microgravity poses numerous challenges to the health of astronauts, and the central nervous system is not exempt. The lack of gravity induces a stress response in NSCs, with a resultant increase in energy production and autophagy-like behavior. This may have important implications in the planning of future space travel, especially for trips of extended duration, to ensure that astronauts have sufficient energy available to sustain CNS homeostasis and to optimize their cognitive and physiological function under times of increased stress.

## Data Availability

The metabolomic raw data will be available upon request.
